# Targeting Signaling Pathways in Cancer Stem Cells for Cancer Treatment

**DOI:** 10.1155/2017/2925869

**Published:** 2017-03-05

**Authors:** Jeffrey Koury, Li Zhong, Jijun Hao

**Affiliations:** ^1^Graduate College of Biomedical Sciences, Western University of Health Sciences, Pomona, CA 91766, USA; ^2^Department of Cell Biology, College of Life Sciences, Hebei University, Baoding, Hebei, China; ^3^Department of Basic Medical Sciences, College of Osteopathic Medicine of the Pacific, Western University of Health Sciences, Pomona, CA, USA; ^4^College of Veterinary Medicine, Western University of Health Sciences, Pomona, CA 91766, USA

## Abstract

The Wnt, Hedgehog, and Notch pathways are inherent signaling pathways in normal embryogenesis, development, and hemostasis. However, dysfunctions of these pathways are evident in multiple tumor types and malignancies. Specifically, aberrant activation of these pathways is implicated in modulation of cancer stem cells (CSCs), a small subset of cancer cells capable of self-renewal and differentiation into heterogeneous tumor cells. The CSCs are accountable for tumor initiation, growth, and recurrence. In this review, we focus on roles of Wnt, Hedgehog, and Notch pathways in CSCs' stemness and functions and summarize therapeutic studies targeting these pathways to eliminate CSCs and improve overall cancer treatment outcomes.

## 1. Introduction

Cancer stem cells (CSCs) are a small subset of cancer cells with the capability of self-renewal and differentiation into heterogeneous tumor cells, and they have been believed to be responsible for tumor initiation, growth, and recurrence. The first population of CSCs was identified in human acute myeloid leukemia (AML), where they displayed strong tumorigenic ability in an in vivo mouse model [1, 2]. Subsequently, many laboratories across the globe have been able to capture and propagate CSCs from a variety of human tumors including brain cancer, melanoma and breast cancer, liver cancer, pancreatic cancer, colon cancer, and prostate cancer [3–9]. As CSCs can survive traditional cancer therapies and result in tumor recurrence and drug resistance [10–12], eradication of CSCs in tumors may represent an effective anticancer therapeutic strategy. Towards this goal, significant efforts have been made to explore the signaling mechanisms underlying CSCs' self-renewal and differentiation, as well as development of regimens targeting the CSCs. In this review, we focus on three key evolutionarily conserved CSC signaling pathways (Wnt, Hedgehog, and Notch pathways) and therapeutic strategies disrupting CSCs' stemness and functions by modulating these pathways.

## 2. Signaling Pathways in CSCs

**Figure 1:**
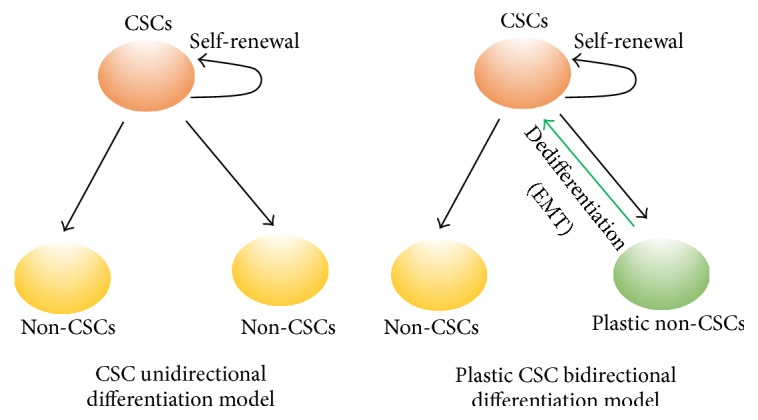
A schematic outlining the classical CSC unidirectional differentiation model and the plastic CSC bidirectional dedifferentiation model. In the unidirectional differentiation model, CSCs preexist in the tumor environment and solely self-renew or differentiate into the non-CSC tumor cells. However, in the plastic CSC bidirectional model, the plastic non-CSC tumor cells can dedifferentiate to acquire a CSC phenotype via epithelial-mesenchymal transition (EMT).

In the past, multiple CSC models have been proposed for tumor heterogeneity including the classical CSC unidirectional differentiation model and the plastic CSC bidirectional dedifferentiation model [13, 14] (Figure 1). In the classical CSC unidirectional differentiation model, CSCs differentiate to non-CSC tumor cells that are unable to move back up the hierarchy to acquire CSC-like activity; however, in the plastic CSC bidirectional dedifferentiation model, non-CSC tumor cells can undergo a dedifferentiation process and acquire CSC-like properties, presumably through epithelial-mesenchymal transition (EMT) in carcinoma [15–19]. Nevertheless, in either CSC model, Wnt, Hedgehog, and Notch pathways are considered important CSCs' regulators.

### 2.1. Canonical Wnt Signaling Pathway

**Figure 2:**
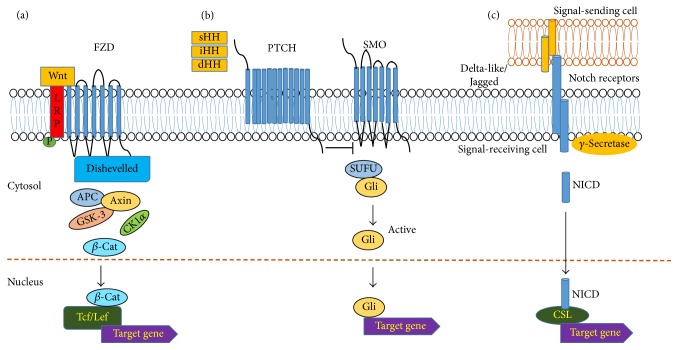
Wnt, Hedgehog, and Notch canonical signaling pathways. (a) In the canonical Wnt signaling pathway, when Wnt ligand binds to FZD and LRP receptors, the *β*-catenin destruction complex (Axin, GSK3, APC, and CK1) is decomposed, and active *β*-catenin accumulates and translocates to the nucleus for target gene transcription. (b) In the canonical Hedgehog pathway, when ligand binds to PTCH, the inhibitory effect of PTCH to SMO is removed. Subsequently GLI is activated and translocates into nucleus for target gene transcription. (c) In the canonical Notch pathway, delta-like or Jagged ligand binds to Notch receptor and a series of extracellular and intracellular cleavages occur, and NICD translocates to the nucleus to regulate target gene transcription. For the detailed pathway information, please refer to the text.

Canonical Wnt pathway, in which Wnt ligands signal through *β*-catenin for their biological functions, is a critical evolutionarily conserved pathway in embryonic development and tissue homeostasis [20]. In the absence of Wnt ligands, the cytoplasmic *β*-catenin is phosphorylated for proteasome-dependent degradation by a “destruction complex” consisting of axin, adenomatous polyposis coli (APC), glycogen synthase kinase 3*β* (GSK3*β*), and casein kinase I*α* (CKI*α*) [21]. However, in the presence of the Wnt ligands, the signaling is activated through the ligands binding to the seven-transmembrane receptor Frizzled (FZD) and the single-membrane-spanning low-density receptor-related protein 5/6 (LRP5/6). FZD then recruits the intracellular protein dishevelled (Dvl), which subsequently sequesters Axin and GSK3*β* from the cytoplasm to the cellular membrane resulting in decomposition of the “destruction complex” [22]. Consequently, the active unphosphorylated *β*-catenin accumulates and translocates into the nucleus to regulate target gene expression (Figure 2(a)).

#### 2.1.1. Abnormal Wnt Signaling Activation and CSCs

Abnormal activation of Wnt signaling has been implicated in the regulation of a plethora of CSC types including colorectal cancer, breast cancer, hematologic cancer, skin cancer, and lung cancer [23–27]. For instance, in colorectal cancer, Wnt signaling deregulation is often associated with mutations in APC and *β*-catenin genes [28]. Mutations in APC have been found in around 80% of all human colon tumors, and these mutations inactivate APC function resulting in Wnt signaling activation by preventing *β*-catenin phosphorylation and subsequent *β*-catenin degradation [29, 30]. In addition, *β*-catenin oncogenic mutations have been reported in approximately 10% of colorectal cancer patients, and these missense or deletion mutations are located at *β*-catenin sites where GSK3*β* normally phosphorylates *β*-catenin, leading to stable *β*-catenin translocation into the nucleus for Wnt activation [22, 31]. Abnormal activation of Wnt signaling disrupts the normal growth and differentiation of colonic crypt stem cells, resulting in a colorectal CSC phenotype by upregulating expression of target genes such as* c-myc* and cyclin D [22, 32]. Moreover, in a recent comparative analysis of signaling pathways between the CD^44+^/CD^133+^ colorectal CSCs and CD^44−^/CD^133−^ cancer cells, Wnt pathway was shown to be highly associated with CD^44+^/CD^133+^ colorectal CSCs [33].

In addition to colorectal CSCs, Wnt signaling is also involved in other types of cancer CSCs. For example, in an elegant study of squamous cell carcinomas, canonical Wnt signaling activation was shown to be critical in tumorigenesis of CD^34+^ bulge CSCs, and ablation of the *β*-catenin gene resulted in depletion of CD^34+^ CSCs and complete tumor regression in mice [27]. In addition, *β*-catenin-deficient tumor cells devoid of the CD^34+^ CSCs were unable to propagate secondary tumors, and conversely tamoxifen-induced expression of a nondegradable *β*-catenin in the skin sufficiently expanded the bulge CSC's population [27].

Moreover, dedifferentiation through EMT is a critical step for non-CSC tumor cells to acquire CSC-like properties as defined by the plastic CSC bidirectional model. Wnt signaling plays an important role in this cancer cell dedifferentiation. One study showed that experimental knockdown of CD146 can dedifferentiate colorectal cancer cells to acquire a stem cell phenotype through inhibiting GSK-3*β* which in turn promoted nuclear translocation of *β*-catenin for Wnt signaling activation [34]. Therefore, modifying Wnt signaling may be essential in the pursuit to curb colorectal cancer, specifically colorectal cancer stem cells.

#### 2.1.2. Therapeutic Agents Targeting Wnt Signaling

As Wnt signaling activation is implicated in CSC's self-renewal, tumorigenesis, and cancer cell dedifferentiation into CSCs, targeting CSCs by inhibiting the Wnt signaling may be a promising therapeutic approach for cancer. Recently numerous Wnt signaling inhibitors, including biological agents and small molecule agents, have been developed [35]. However, to date, no Wnt signaling inhibitors have been approved for clinical usage. The majority of Wnt inhibitors have been evaluated preclinically, and the readers can refer to our recent review paper in this area [35]. Here we summarize ongoing clinical trials of Wnt inhibitors (Table 1). For instance, one clinical trial of a Wnt inhibitor is PRI-724 which inhibits the Wnt signaling by specifically binding to downstream CREB-binding protein. PRI-724 was previously shown to induce apoptosis of colon carcinoma cells and exhibit antitumor activity in the mouse xenograft models of colon cancer [36]. In the phase I trial, 18 patients were treated showing favorable toxicity profiles with only one dose-limiting toxicity of grade 3 of reversible hyperbilirubinemia [37]. An impending phase II trial for PRI-724 is almost underway involving mFOLFOX6/Bevacizumab with or without PRI-724. The focus of the study is to target patients with Stage IV metastatic colorectal cancer (NCT Number: NCT02413853). In addition, Porcupine is a membrane bound O-acyltransferase (MBOAT) specific to Wnt posttranslational acylation, which is required for subsequent Wnt secretion, and loss of Porcupine can lead to Wnt signaling inhibition [38, 39]. In addition, a specific small molecular Porcupine inhibitor LGK974 was identified in a luciferase-based cell screening and was efficacious in targeting Wnt signaling in multiple tumor models including murine and rat mechanistic breast cancer models and a human head and neck squamous cell carcinoma model [40]. Recently, a phase I, open label dose escalation trial of the LGK974 has been initiated to treat a variety of malignancies including melanoma, breast cancer, and pancreatic adenocarcinoma (NCT Number: NCT01351103). Other than small molecules, several biologic therapeutic agents targeting the Wnt pathway have entered clinical trials as well (Table 1). For instance, OMP18R5 (also known as Vantictumab), a fully humanized monoclonal antibody that targets FZD receptor, has recently concluded an open label phase 1 dose escalation study for solid tumors [41, 42] (NCT Number: NCT01345201). Another biologic therapeutic agent OMP-54F28, a fusion protein that binds Wnt ligands and prevents them from binding to FZD receptors, was recently developed. In phase I trial of OMP-54F28, minimum doses at 0.5 mg/kg ranging up towards 10 mg/kg were administered intravenously once every 3 weeks in patients with solid tumors (NCT Number: NCT01608867). Although this clinical trial has recently been completed, the results have not been announced publicly [41, 42]. Moreover, another two phase 1b trials are underway involving OMP-54F28 and the chemotherapy drug Paclitaxel to treat ovarian and Stage IV pancreatic cancer (NCT Numbers: NCT02092363, NCT02050178).

### 2.2. Hedgehog Signaling

HH signaling is essential in a wide variety of cellular and molecular processes during embryogenesis, development, and adult tissue homeostasis [43, 44]. Three Hedgehog homologues, Sonic Hedgehog (sHH), Indian Hedgehog (iHH), and Desert Hedgehog (dHH), have been well studied in mammals [45, 46]. In the absence of HH ligands, a cell-surface transmembrane protein Patched (PTCH) inhibits the transmembrane protein Smoothened (SMO), and full length GLI proteins are then proteolytically processed to generate the repressor GLI^R^ to suppress HH signaling target gene expression (Figure 2). However, when extracellular HH ligands bind to PTCH, PTCH's inhibitory influence on SMO is removed, and activation of SMO results in nuclear translocation of GLI and induction of HH signaling target gene transcription [45, 47].

#### 2.2.1. Abnormal HH Signaling Activation and CSCs

Aberrant activation of the HH pathway in CSC's regulation and maintenance has been reported in numerous cancer types including glioblastoma, lung squamous cell carcinoma, breast cancer, pancreatic adenocarcinoma, myeloma, and chronic myeloid leukemia (CML) [48–53]. In multiple myeloma CSCs, it was shown that SMO and Gli1 were highly expressed in comparison to non-CSCs, and activation of HH signaling by HH ligands promoted multiple myeloma CSC's expansion, whereas inhibition of the HH signaling markedly blocked CSC's clonal expansion [54]. The data supports that HH signaling promotes multiple myeloma CSC's functions. Moreover, higher activation of HH signaling was also observed in CSCs of human lung squamous cell carcinoma and glioma as compared to bulk tumor cells, further supporting aberrant HH signaling activation's critical role for CSC self-renewal and regulation [49, 53]. In another study, Zhao et al. reported that HH pathway activation was also involved in maintenance of CML CSCs [52]. In a murine CML model of study, deletion of SMO significantly reduced the CML CSCs, and conversely overexpression of SMO in a SMO-deficient mouse CML model dramatically enhanced CML CSCs 4-fold and significantly increased CML progression [52]. In addition, inhibition of HH signaling by SMO antagonist cyclopamine reduced a glioblastoma stem cells population [50], and similar findings have been observed in colon CSCs, pancreatic CSCs, prostate CSCs, and lung CSCs [55–58].

In the plastic CSC bidirectional dedifferentiation model, HH signaling plays an important role during the EMT process to acquire stem cell-like phenotypes. For instance, Gli1 was shown to correlate with markers of EMT and highly express in the claudin-low breast CSCs, and knockdown of Gli1 resulted in reduced claudin-low breast CSC's viability, motility, clonogenicity, and self-renewal as well as tumor growth in orthotopic xenografts [59]. Recently, Wang et al. have demonstrated that HH pathway and EMT are active in pancreatic cancer cells-derived tumorspheres that exhibit CSC properties, and inhibition of HH signaling by SMO knockdown blocks the self-renewal, EMT, invasion, chemoresistance, and tumorigenesis of pancreatic CSCs [60].

#### 2.2.2. Therapeutic Agents Targeting HH Signaling

As evidenced above, HH signaling plays a critical role in CSC's self-renewal and regulation, and inhibition of the HH pathway disrupts CSC's stemness and induces CSC's differentiation which are desirable for cancer treatment. In the past, numerous HH pathway inhibitors have been developed. For a complete review of HH signaling inhibitors, please refer to the recent reviews [61–63]. Here we only summarize the HH signaling inhibitors either approved by FDA or under clinical trials (Table 2). Vismodegib developed by Genentech is the first HH signaling inhibitor approved by FDA. Vismodegib targets SMO for HH signaling inhibition and has been used to treat metastatic basal cell carcinoma. The initial phase I trial of Vismodegib showed that 18 out of 33 enrolled patients with locally advanced or metastatic tumors had a response to Vismodegib. Among the remaining 15 patients, 11 had stable disease for up to 10.8 months and 4 had progressive disease. No dose-limiting toxic effects or grade 5 adverse events were seen in the trial and reported toxicities were mild with common side effects of mild loss of taste, hair loss, weight loss, and hyponatremia [64, 65]. Currently, clinical trials of Vismodegib as a monotherapy or in combination with other therapeutic drugs are ongoing for various cancers including medulloblastoma, small cell lung cancer, metastatic pancreatic cancer, metastatic prostate cancer, intracranial meningioma, recurrent glioblastoma, and acute myeloid leukemia (NCT Numbers: NCT00833417, NCT01201915, NCT00739661, and NCT01088815) [61]. In 2015, a new SMO inhibitor, Sonidegib, was approved by the FDA to treat adult patients with locally advanced BCC. In the dose escalation phase I trial, maximum tolerated doses of 800 mg daily and 250 mg twice daily were established [66]. Grade 1/2 adverse effects were apparent, consisting of nausea, anorexia, vomiting, muscle spasms, fatigue, and alopecia and grade 3/4 adverse effects were weight loss, hyperbilirubinemia, myalgia, fatigue, and dizziness (NCT Number: NCT01529450) [66]. A phase II study demonstrated that Sonidegib sustained tumor responses in patients with advanced BCC after a 12-month follow-up [67]. Currently several phase I/II trials of Sonidegib to treat other solid tumors and hematological malignancies are still underway (NCT Numbers: NCT02195973, NCT01487785) [61]. Moreover, a few of HH signaling inhibitors that are actively being tested in clinical trials include additional SMO inhibitors (Saridegib, BMS-833923, Glasdegib, and PF-5274857) and GLI Inhibitor (arsenic trioxide). Other than small molecule inhibitors, a monoclonal antibody, 5E1, blocks binding of all three mammalian HH ligands to PTCH for HH signaling inhibition [68, 69]; however, this antibody has not entered clinical trials yet.

### 2.3. Canonical Notch Signaling

Canonical Notch signaling is another essential evolutionarily conserved pathway in development and adult tissue homeostasis [70]. Notch signaling is activated when the extracellular domain of Notch transmembrane receptor binds to Notch ligands and subsequently induces proteolytic cleavage and release of the intracellular domain (Notch ICD or NICD) of Notch. The Notch ICD then translocates to the nucleus where it interacts with a CBF1/Suppressor of Hairless/LAG-1 (CSL) family DNA-binding protein and regulates the expression of target genes including those pertinent to CSC self-renewal such as Survivin, Myc Nanog, Oct-4, and Sox2 (Figure 2) [71–76]. In mammals, four Notch receptors (Notch 1–Notch 4) and five ligands (Jagged 1 and 2 and Delta-like 1, 3, and 4) have been identified [77].

#### 2.3.1. Abnormal Notch Signaling Activation and CSCs

Abnormal activation of Notch signaling plays a pivotal role in the CSCs of breast cancer, pancreatic cancer, and glioblastoma. For instance, Barnawi et al. reported that fascin (an actin-bundling protein) effectively regulates breast CSCs at least partially through Notch pathway [78]. Fascin knockdown significantly reduced breast stem cell-like phenotype (downregulation of stem cell pluripotent genes such as Oct4, Nanog, Sox2, and Klf4), and the cells became less competent in forming colonies and tumorspheres. Conversely, activation of Notch signaling induced the relevant downstream targets predominantly in the fascin-positive cells, and fascin-positive CSCs showed stronger tumorigenesis [78]. In another study, immunohistochemical analysis of 115 breast tumor tissues from primary lesions was performed, and results showed that Notch positive tissues were significantly associated with a CSC marker aldehyde dehydrogenase 1 family member A1 levels [79]. Very recently, Choy et al. reported that Notch 3 signaled constitutively in a panel of basal breast cancer cell lines and in more than one-third of breast basal tumors [80].

Moreover, the important role of Notch signaling was also demonstrated in several other types of CSCs. In a study of patient-derived pancreatic CSCs, Notch ligands Notch 1, Notch 3, Jag1, Jag2, and Notch target gene Hes1 were found to be highly expressed in the pancreatic CSCs, and an inhibitor of *γ*-secretase (an important protease mediating Notch signaling by releasing the Notch ICD) significantly decreased the CSC's subpopulation and tumorsphere formation [81]. Moreover, activation of Notch signaling by delta/Serrate/Lag-2 peptide or inhibition of the signaling by knockdown of Hes1 enhanced or decreased pancreatic CSC's tumorsphere formation, respectively [81]. In addition, Notch signaling dysregulation has also been recognized in glioblastoma CSCs [82]. It was found that Protein Kinase C Iota (PKCi) was highly expressed in glioblastoma patient-derived CSCs, and silencing PKCi resulted in apoptosis and reduction of proliferation of the glioblastoma CSCs in vitro and tumor growth in vivo in a xenograft mouse model [83]. Gene expression profiling of PKCi-silenced glioblastoma CSCs revealed a novel role of the Notch signaling pathway in PKCi mediated glioblastoma CSC's survival [83]. In addition to its important roles in CSCs, Notch signaling is also involved in EMT to promote cancer cell acquisition of a stem-like phenotype and drug resistance. For instance, prostate cancer cells undergoing EMT displayed stem-like cell features characterized by increased expression of Notch 1 and other pluripotent genes such as Sox2, Nanog, Oct4, and Lin28 [84].

#### 2.3.2. Therapeutic Agents Targeting Notch Signaling

Therapeutics targeting the Notch pathway mostly consist of *γ*-secretase inhibitors and anti-DLL4 antibodies (Table 3). Inhibition of the Notch pathway via *γ*-secretase inhibitors prevents Notch receptor cleavage at the cell surface, thus blocking activation of self-renewal target genes. In a preclinical study, a *γ*-secretase inhibitor RO4929097 significantly suppressed Notch target genes Hes1, Hey1, and HeyL [85]. Several phase I and phase II studies have been conducted in hopes of synergistically utilizing RO4929097 with other agents for cancer treatment. For instance, in a completed phase I trial, RO4929097 and Cediranib Maleate were used in tandem to determine the phase II dose and safety profile of RO4929097 in solid tumors (NCT Number: NCT01131234), and the clinical trial data shall be announced soon. Another *γ*-secretase inhibitor is LY900009, developed by Eli Lilly, which is in phase I for patients with advanced cancer including leiomyosarcoma and ovarian cancer [86]. A third *γ*-secretase inhibitor (PF-003084014) was developed by Pfizer, and it is progressing in its phase I trials in patients with T-cell acute lymphoblastic leukemia and T-cell lymphoblastic lymphoma [87]. In addition to *γ*-secretase inhibitors, another category of Notch pathway molecules is monoclonal antibodies that target DLL4 (Delta-like ligand 4) to prevent ligand binding. Enoticumab (REGN421) is an anti-DLL4 antibody that has been used to target advanced solid tumors with overexpression of DLL4 (such as ovarian cancer) [88]. In 2015, a recommended phase II dose of 4 mg/kg every 3 weeks or 3 mg/kg every 2 weeks administered intravenously was established based on PK profiles in patients diagnosed with ovarian, colon, or breast cancer. [89]. Another anti-DLL4 monoclonal antibody developed by OncoMed Pharmaceuticals and Celgene is Demcizumab, which has recently completed a phase I dose escalation clinical trial as well. In this study, Demcizumab was well tolerated at doses ≤5 mg with disease stabilization and tumor size decreases when administered weekly. The side effects of Demcizumab include hypertension and an increased risk of congestive heart failure in prolonged drug administration (NCT Number: NCT02722954) [90].

## 3. Crosstalk among Pathways and Combination Treatments

Many pathways do not act as isolated units but rather often interact with other pathways as a biological network during development and homeostasis. Crosstalk among Wnt, HH, Notch, and other pathways have been reported in cancer and CSCs [91]. For instance, in a colorectal cancer study, progastrin secreted by colorectal tumors was shown to activate Wnt signaling and result in expression of Wnt target genes including Jagged-1, one Notch ligand. Upregulation of Jagged-1 induces Notch signaling which in turn may further elevate *β*-catenin activity of progastrin-driven Wnt and Notch signaling in colorectal cancer cells [92]. Similarly, in breast CSCs, Mel-18 was reported as a negative regulator of breast CSC's self-renewal. Knockdown of Mel-18 increased Wnt signaling, which subsequently upregulated Wnt target gene jagged-1's expression, leading to activation of the Notch pathway for CSC's self-renewal [93]. In addition, HH signaling can crosstalk with both Wnt and Notch pathways as well. In gastric cancer cells, HH signaling was shown to suppress Wnt signaling through the soluble frizzled-related protein 1 (sFrP1), a target gene of HH signaling capable of modulating Wnt pathway by directly binding to Wnt ligands [94]. In another study of glioblastoma cells and patient specimens, Notch signaling inhibition was shown to downregulate its target gene Hes1 which in turn upregulates GLI transcription in the HH pathway [95].

Complex signaling networks are known to contribute to the cellular diversity of stem cells during embryogenesis and tissue homeostasis and may play essential roles in the cancer and CSC's biology. In recent years, significant efforts have been made to develop combination therapies to target multiple signaling pathways for cancer treatments. For instance, a recent study demonstrated that combination inhibition of both Notch and HH signaling depleted the CSC subpopulation cells in a prostate cancer model [96]. In addition, a clinical trial of combination of HH pathway inhibitor Vismodegib and Notch signaling inhibitor RO4929097 has been conducted in patients with advanced breast and sarcoma. In another recent study, Sharma et al. showed that combination treatment with HH signaling inhibitor NVP-LDE225 and pI3/mTOR/Akt signaling inhibitor NVP-BEZ235 inhibited self-renewal capacity of pancreatic CSCs by suppressing the expression of pluripotency maintaining factors Nanog, Oct-4, Sox-2, and c-Myc and transcription of GLI [97].

## 4. Conclusions

Since the first identification of CSCs in leukemia, the important roles of CSCs in cancer progression, metastasis, and relapse as well as drug resistance have been increasingly recognized. Eradication of CSCs by targeting the key signaling pathways underlying CSC's stemness and function represents a promising approach in cancer treatment. In this review, we mainly summarized the three critical evolutionarily conserved pathways (Wnt, HH, and Notch signaling) in CSCs and potential therapies targeting these pathways for cancer treatment. To date, numerous agents have been developed to specifically target each of these pathways for cancer treatments. Nevertheless, it has been recognized that the signaling pathways may interact with each other as a coordinated network to regulate CSC stemness and functions. Therefore, understanding the crosstalk among the signaling pathways in CSC regulation is critical for the development of therapies targeting CSCs.

## Figures and Tables

**Table 1:** Small molecule/biological therapeutics targeting the Wnt pathway.

Molecule	Function	Phase/clinical trials	Cancer type	NCT Number
PRI-724	Dishevelled inhibitor	Phase 1/2	Pancreatic cancer, acute myeloid leukemia, and colon cancer	NCT02413853 NCT01606579 NCT01764477

LGK974	Porcupine inhibitor	Phase 1	Melanoma, breast cancer, and pancreatic adenocarcinoma	NCT01351103

Vantictumab (OMP18R5)	Anti-Frizzled 7 receptor	Phase 1b	HER2 negative breast cancer and pancreatic cancer	NCT01345201

Ipafricept (OMP-54F28)	Fc-Frizzled 8 receptor	Phase 1a/1b	Pancreatic cancer and ovarian cancer	NCT02092363 NCT02050178

The clinical trial information was accessed via https://clinicaltrials.gov with National Clinical TrialNumber (NCT Number).

**Table 2:** Small molecule/biological therapeutics targeting the Hedgehog pathway.

Molecule	Function	Phase/clinical trials	Cancer type	NCT Number
GDC-0449 (Vismodegib derivative)	SMO inhibitor	Phase 2	Basal cell carcinoma, ovarian cancer, metastatic pancreatic cancer, medulloblastoma, small cell lung cancer, metastatic prostate cancer, glioblastoma, and acute myeloid leukemia	NCT00833417 NCT01201915 NCT00739661 NCT01088815

Genistein	Downregulate Gli1	Phase 1/2	Colorectal cancer	NCT01985763

Sonidegib (LDE225)	SMO inhibitor	Phase 1/2	Prostate cancer, ovarian cancer, pancreatic cancer, and basal cell carcinoma	NCT02195973 NCT01487785 NCT01529450

5E1	Prevent HH ligand-Patched binding	Preclinical	Prostate cancer	N/A

Glasdegib (PF-04449913)	SMO inhibitor	Phase 1b/2	Acute myeloid leukemia, chronic myelomonocytic leukemia	NCT01841333 NCT01286467

The clinical trial information was accessed via https://clinicaltrials.gov with National Clinical TrialNumber (NCT Number).

**Table 3:** Small molecule/biological therapeutics targeting the Notch pathway.

Molecule	Function	Phase/clinical trials	Cancer type	NCT Number
RO4929097	Gamma secretase inhibitor	Phase 2	Breast cancer, ovarian cancer, renal cell carcinoma	NCT01131234

LY900009	Gamma secretase inhibitor	Phase 1	Advanced solid tumor or lymphoma	NCT01158404

PF-03084014	Gamma secretase inhibitor	Phase 1/2	T-cell acute lymphoblastic leukemia and T-cell lymphoblastic lymphoma	NCT00878189 NCT01981551

Enoticumab	Anti-DLL4 antibody	Phase 1	Advanced solid tumors and ovarian cancer	NCT00871559

Demcizumab	Anti-DLL4 antibody	Phase 1b/2	Advanced solid tumors, pancreatic cancer, ovarian cancer, and non-small cell lung cancer	NCT02722954 NCT01189968 NCT01189929

Tarextumab	Anti-Notch 2/3	Phase 1b/2	Solid tumors, Stage IV pancreatic cancer, and Stage IV small cell lung cancer	NCT01277146 NCT01647828 NCT01859741

The clinical trial information was accessed via https://clinicaltrials.gov with National Clinical TrialNumber (NCT Number).
